# Interferon-alpha promotes immunosuppression through IFNAR1/STAT1 signalling in head and neck squamous cell carcinoma

**DOI:** 10.1038/s41416-018-0352-y

**Published:** 2018-12-17

**Authors:** Hailong Ma, Wenyi Yang, Liming Zhang, Shuli Liu, Mei Zhao, Ge Zhou, Lizhen Wang, Shufang Jin, Zhiyuan Zhang, Jingzhou Hu

**Affiliations:** 1 Department of Oral Maxillofacial-Head and Neck Oncology, Shanghai Ninth People’s Hospital, Shanghai Jiao Tong University School of Medicine, Shanghai, 200011 China; 2 Shanghai Key Laboratory of Stomatology & Shanghai Research Institute of Stomatology, National Clinical Research Center of Stomatology, Shanghai, 200011 China; 3 Department of Head and Neck Surgery, The University of Texas MD Anderson Cancer Center, Houston, TX 77030 USA; 4 Department of Oral Pathology, Shanghai Ninth People’s Hospital, Shanghai Jiao Tong University School of Medicine, Shanghai, 200011 China

**Keywords:** Cancer microenvironment, Cell signalling

## Abstract

**Background:**

An immunosuppressive microenvironment is critical for cancer initiation and progression. Whether interferon alpha (IFNα) can suppress immune and cancer cells and its involved mechanism still remain largely elusive.

**Methods:**

We examine the expression of interferon alpha/beta receptor-1 (IFNAR1), CD8, CD56 and programmed death ligand 1 (PDL1) in head and neck squamous cell carcinomas (HNSCC). The effect of IFNα on PDL1 and programmed cell death protein 1 (PD1) expression in tumour cells and immune cells was detected in vitro and in vivo.

**Results:**

Overexpression of IFNAR1, MX1 and signal transducer and activator of transcription 1 (Stat1) indicated the endogenous IFNα activation in tumour microenvironment, which correlated with immunosuppression status in HNSCC patients. Moreover, IFNα transcriptionally activated the expression of PDL1 through p-Stat1 (Tyr701) and promoted PD1 expression in immune cells through IFNAR1. The inhibition of IFNα signalling enhanced the cytotoxic activity of nature killer cells. At lastastly, we confirmed the upregulation of PDL1 and PD1 in response to IFNα treatment in both xenograft tumour models and patient-derived xenograft models.

**Conclusions:**

Our findings demonstrate that IFNα-induced PDL1 and PD1 expression is a new mechanism of immunosuppression in HNSCC, suggesting that blocking IFNα signalling may enhance the efficacy of immune checkpoint blockade.

## Introduction

Head and neck squamous cell carcinoma (HNSCC) accounts for ~ 90% of head and neck cancer. It has poor prognosis and often results in serious physiologic and psychological complications after traditional therapies.^1,2^ In recent years, substantial advances have been made in the development of therapeutic approaches for HNSCC, including targeted therapies, immunotherapy, and immune checkpoint blockade (ICB) in particular.^3^ Targeted therapies specific for programmed cell death 1 (PD1) and programmed cell death ligand 1 (PDL1) have shown surprising results for recurrent and/or metastatic HNSCC (R/M-HNSCC) in the CheckMate-141^4^ and KEYNOTE-012 trials.^5^ However, ICBs using nivolumab and pembrolizumab only resulted in a modest overall response rate of ~ 15% in second-line treatment.^6^ Moreover, immune-related adverse events, especially high-grade gastrointestinal and liver toxicities, have also directly impacted clinical outcomes.^7^ So, understanding of mechanisms involved in immunosuppression mediated by PDL1 and PD1 is very critical for improving therapeutic efficacy of ICBs in HNSCCs.

Interferon alpha (IFNα) is a pleiotropic cytokine belonging to the type I IFN family that is originally described for its antiviral activity.^8^ IFNα is produced by most nucleated cells, and its signalling is mediated through a receptor complex composed of two subunits, interferon alpha/beta receptor-1 (IFNAR1) and IFNAR2.^9^ IFNAR1 has a very weak ligand binding affinity, but it induces intracellular signalling cascades to create a docking site for signal transducers and activators of transcription (STATs).^10^ Upon IFNα stimulation, heterodimers of Stat1 and Stat2 translocate to the nucleus to induce the expression of IFN-stimulated genes.^11^ IFNα can also activate other members of the STAT family, such as Stat3, Stat4, Stat5 and Stat6.^12^ It has been reported that interferon signalling was constitutively activated and that it promoted the immune evasion of glioma cells.^13^ Our previous study revealed that IFNα had a synergistic antitumour effect with epidermal growth factor receptor-targeting therapies in HNSCC.^14^ Whether IFNα is constitutively activated in tumour microenvironment of HNSCC and whether it can promote immunosuppression in HNSCC are still unclear.

In this study, we examined the expression of IFNAR1, CD8, CD56 and PDL1 in human HNSCC tissues, and our results showed that the overexpression of IFNAR1 was significantly associated with the immunosuppressive status in HNSCC. Moreover, we demonstrated an IFNα-IFNAR1/STAT1-PDL1/PD1 axis that play an important role in development of immunosuppressive environment in HNSCC, which might help improve the efficacy of ICBs.

## Materials and methods

### Tissue samples

From January 2009 to December 2010, 108 patients with follow-up information and with a 90.8% follow-up rate were included in this study. Patients who had received chemotherapy or radiation therapy before surgery were excluded. The stage of the disease was determined according to the tumour-node-metastasis staging (TNM) system. The histological grading of tumours was in accordance with the degree of differentiation in the World Health Organization histological criteria. Fresh tissues from five HNSCC patients (three from the tongue, one from the buccal mucosa and one from the gum) were obtained and subjected to western blot for detecting of IFNAR1 expression. Fresh gingival tissues were obtained during tooth extraction to be primarily cultured. This study was approved by the Ethics Committee of the Ninth People’s Hospital, Shanghai Jiao Tong University School of Medicine (Shanghai, China), and informed consent was obtained from all patients.

### Immunohistochemistry and immunofluorescence

Immunohistochemistry (IHC) was performed as previously described.^15^ In brief, sections were rehydrated and heated in a water bath at 100 °C with citrate buffer or ethylenediaminetetraacetic acid solution for 20 min for antigen retrieval. The sections were incubated overnight at 4 °C with rabbit polyclonal antibodies against IFNAR1 (Abcam, Cambridge, MA, UK and Sigma-Aldrich, St. Louis, MO, USA), CD8 (Abcam, Cambridge, MA, UK), CD56 (Proteintech, Rosemont, IL, USA), PDL1 (CST, Danvers, MA, USA) and p-Stat1 (Tyr701) (CST, Danvers, MA, USA). Blinded microscopic examination of the immunohistochemical staining was independently performed by two pathologists. Any divergence was resolved by discussion. The intensity of IFNAR1 immunoreaction was scored as follows: 0 = absence of stained cells; 1 = weak staining; 2 = moderate staining; and 3 = strong staining. The immunoreaction score was calculated by multiplying the staining intensity and the percentage of positive cells. HNSCC tissues were divided into high and low groups according to the cutoff value of 150 for IFNAR1 expression. IHC and image analysis were performed to measure and analyse the mean optical density (OD) for PDL1 and p-Stat1 in the animal experiments. MX1 antibody (Proteintech, Rosemont, IL, USA) was used in the immunofluorescence of HNSCC cell lines.

### Real-time PCR

Total RNA from tissues and cultured cells was isolated with TRIzol (Takara, Dalian, China) according to the manufacturer’s protocol. After extraction, RNA was reversely transcribed to cDNA and amplified by real-time PCR. The conditions for real-time PCR were denaturated at 95 °C for 30 s, followed by 40 cycles of annealing/elongation at 95 °C for 5 s and 60 °C for 30 s on an ABI StepOne Plus system using the following specific primers: *GAPDH* forward: 5′-CCTCTGACTTCAACAGCGAC-3′ and reverse: 5′-TCCTCTTGTGCTCTTGCTGGC-3′; *IFNAR1* forward: 5′-AGTGGCTCCACGCCTTTTTA-3′ and reverse: 5′-GCTTGTACGCGGAGAAGGTA-3′; *IFNAR2* forward: 5′-ATAGCCTCCCCAAAGTCTTGA-3′ and reverse: 5′-ATATCCATGGCTTCCAACGGT-3′; *CD274* forward: 5′-AGACCACCACCACCAATTCC-3′ and reverse: 5′-TGGAGGATGTGCC AGAGGTA-3′; *CD279* forward: 5′-CAGTTCCAAACCCTGGTGGT-3′ and reverse: 5′-GGCTCCTATTGTCCCTCGTG-3′.

### Data mining

To determine the expression of IFNAR1, PDL1, CD8 and MX1 in HNSCC, we performed data mining in three publicly available databases, Oncomine, the Gene Expression Omnibus (GEO) at the National Center for Biotechnology Information (NCBI) and The Cancer Genome Atlas (TCGA) (http://www.cbioportal.org/). The differential expression of the *IFNAR1* gene was probed in 22 paired HNSCC and normal tissue samples from the same donors (GDS2520).^16^ The expression of *IFNAR1*, *MX1* and *STAT1* in HNSCC was also assessed in Oncomine.^17–21^ The co-expression of *STAT1* and *CD274*, *MX1*, *CD279* was assessed in HNSCC samples from TCGA database.^22,23^ Kaplan–Meier analyses of the survival probability of HNSCC patients in TCGA were performed according to the expression of IFNAR1, CD8 and PDL1.

### Cell culture

The cell lines used in this study were SCC4, Cal27, HN4, HN6 and HN30. SCC4 and Cal27 were purchased from ATCC (Manassas, VA). The cell lines HN4 and HN6 were established from tongue squamous carcinoma, whereas HN30 was established from pharyngeal squamous cell carcinoma. HN4, HN6 and HN30 cell lines were kindly provided by the University of Maryland Dental School, USA. All these cell lines were cultured in Dulbecco’s modified Eagle’s medium (DMEM) (Gibco, Carlsbad, CA) and DMEM/F12 (for SCC4) supplemented with 10% fetal bovine serum, 1% glutamine, and 1% penicillin–streptomycin. The cells were cultured in a humidified atmosphere containing 5% CO_2_ at 37 °C. All cell lines were passaged, at most, 15 times between freeze–thaw cycles and routinely screened for mycoplasma. Normal oral keratinocyte (NOK) was cultured from healthy gingiva after tooth extraction. Authentication of cell lines was done by the Characterized Cell Line Core Facility at the Ninth People’s Hospital, Shanghai Jiao Tong University School of Medicine by the STR Method.

### RNA interference-mediated gene silencing

For cell transfection, HNSCC cells were seeded in a six-well plate and transfected with 100 nm small interfering RNA (siRNA) using Lipofectamine^TM^ 3000 (Invitrogen, Carlsbad, CA) according to the manufacturer’s instructions. The sequences of IFNAR1-specific siRNAs are #1, 5′-CAUUUCGCAAAGCUCAGAUdTdT-3′ and #2, 5′-CCAUAUCUAUAUCGGUGCUdTdT-3′. The sequence of the STAT1-specific siRNA is 5′-CGGCUGAAUUUCGGCACCUdTdT-3′. The sequence of the scrambled control is 5′-UUCUCCGAACGUGUCACGUdTdT-3′.

### MTT and CCK8 assay

HNSCC cells were seeded in 96-well plates at 2~5 × 10^3^ cells per well. IFNα was administered at the indicated concentration after cell adherence. After incubation for 72 h, 20 μl MTT (3-(4, 5-dimethylthiazol-2-yl)-2, 5-diphenyltetrazolium bromide) was added into each well and incubated for 4 h. Then, 200 μl DMSO was used to dissolve the formazan crystals in each well. The OD was measured at 490 nm within 10 min. In total, 10 μl CCK8 (Dojindo, Kumamoto, Japan) was added into each well. The OD value was measured at 450 nm with 1~4 h of incubation.

### Flow cytometry

Flow cytometry was performed as previously described.^24^ in brief, HN4 and HN30 cells were incubated with the indicated agent for 48 h. The cells were collected and incubated with anti-human PDL1 antibody at 1:100 (BD Biosciences, Franklin Lakes, NJ) for 30 min on ice. Then, the cells were resuspended in 100 μl fluorescence-activated cell sorting buffer and analysed on BD Fortessa flow cytometer. The final results were analysed with FlowJo software. Signal intensity was calculated as the ratio of the median fluorescence of the PDL1 antibody to that of the isotype control antibody (SFI: specific fluorescence index). CD4-FITC antibody, CD8-PerCP-Cy5.5 antibody, CD56-APC antibody, and PD1-PE antibody (all purchased from BD Biosciences) were applied to detect the PD1 expression on the surface of immune cells from peripheral blood of HNSCC patients and healthy controls. The IFNAR1 antibody (Abcam, Cambridge, MA, UK) and PE-conjugated secondary antibody (Proteintech, Rosemont, IL, USA) were used to analyse the surface IFNAR1 expression on immune cells.

### Western blot

Western blot was performed as previously described.^25^ Antibodies against Stat1, p-Stat1 (pTyr701), Stat3, p-Stat3 (Tyr705) and PDL1 (CST, Danvers, MA) were used in this study. The antibody against IFNAR1 was purchased from Abcam (Abcam, Cambridge, MA, UK). Antibodies against GAPDH, α-tubulin and β-actin (all purchased from Proteintech company, Rocky Hill, NJ, USA) were used as internal controls. The immunoreactive bands were scanned and analysed by using Odyssey Infrared Imaging System (LI-COR Biosciences, Lincoln, NE) and Image J software (NIH, Bethesda, MD).

### Isolation of peripheral blood mononuclear cells (PBMCs) and the purification of immune cells

Approximately 5 ~ 10 ml peripheral blood was obtained from healthy controls and HNSCC patients. PBMCs were isolated by a density gradient using Ficoll-Paque PLUS (GE, Uppsala, Sweden) following the manufacturer’s instructions. The average cell number was between 0.5~1.2 × 10^9^ PBMCs. CD4^+^ T, CD8^+^ T and CD56^+^ natural killer (NK) cells were enriched by magnetic cell sorting (Miltenyi Biotec, Bergisch Gladbach, Germany) of freshly isolated PBMCs using magnetic beads labelled with CD4^−^, CD8^−^, CD56-specific antibodies (Miltenyi Biotec, Bergisch Gladbach, Germany) following manufacturer’s instructions. The purity of the enriched cells was > 95% as assessed by flow cytometry. Freshly isolated PBMCs or enriched CD4^+^ T, CD8^+^ T and NK cells were cultured in RPMI 1640 (Gibco, Waltham, MA) supplemented with 10% fetal bovine serum, 1% glutamine, and 1% penicillin–streptomycin. The cells were cultured in a humidified atmosphere containing 5% CO_2_ at 37 °C.

### NK cell lysis, granzyme M and perforin release assays

NK cells lysis assays were performed as previously described.^26^ After being transfected for 48 h, HN4 and HN30 cells were seeded in 96-well plates at 1~3 × 10^3^ cells per well. The adherent cells were co-cultured with NK cells at different effector-to-target (E:T) cell ratios as indicated for 4 h. The viability of tumour cells was measured with luciferase assay.

The release of granzyme M (GZMM) and perforin (PF) of NK cells were measured by GZMM-enzyme-linked immunosorbent assay (ELISA) kit and PF-ELISA kit (Ybio, Shanghai, China). In the NK cells group, 1 × 10^5^ NK cells (control and treated with 10 μg/ml recombinant PDL1 protein (Sino Biological, Shanghai, China)) were seeded into 96-well plate. In the co-culture group, 1 × 10^4^ tumour cells were seeded into 96-well plate with 1 × 10^5^ NK cells. After culture for 24 h, the supernatant culture medium were collected and analysed by ELISA kit. The luciferase assay and ELISA were read at 450 nm by the Spectra Max i3 (Molecular Devices, Bedford, MA, USA).

### Chromatin immunoprecipitation (ChIP)

ChIP was strictly performed according to the protocol of the SimpleChIP Enzymatic Chromatin IP kit (purchased from CST) as our previous study.^27^ In brief, after treatment with 100 ng/ml IFNα for 48 h, HN4 and HN30 cells were fixed with 1% formaldehyde for 10 min, which was then quenched with glycine for 5 min at room temperature. The cell lysate was digested with micrococcal nuclease at 37 °C for 20 min and sonicated at 30% output for 6 × 10 sec to obtain specific nucleotide fragments (150~900 bp). After incubation with anti-p-Stat1 (Tyr701) antibody (CST, Danvers, MA) or normal rabbit IgG overnight at 4 °C with rotation, and then 30 μl of ChIP-grade Protein G magnetic beads was added and incubated for 2 h at 4 °C with rotation. Quantitative polymerase chain reaction analysis of purified ChIP DNA (ChIP-qPCR) was performed to calculate the percentage enrichment of promoter regions using the 2^-ΔΔCT^ method. The primers sequences specific for the *CD274* promoter are forward, 5′-ATCTCATTTACAAGAAAACTGGACTGAC-3′ and reverse, 5′-AGGCCCGGAGGCGGG-3′.

### Luciferase reporter assay

Cells were seeded in 12-well plates (1 × 10^5^ cells/well) and grown to 40–50% confluence. *CD274* promoter-luciferase plasmids (constructed by Obio Technology (Shanghai) Corporations) were co-transfected into cells with the help of pRL-TK (TK promoter Renilla luciferase construct as the internal control). HN4 and HN30 cells were transiently transfected using Lipofectamine^TM^ 3000 transfection reagent (Invitrogen, Carlsbad, CA, USA). Then, the indicated concentrations of IFNα were added at 24 h after transfection. Luciferase activity was determined at 24 h after stimulation using a Dual-luciferase Reporter Assay System (Beyotime, Shanghai, China). In brief, cell lysates (200 μl/well) were used to measure the relative luciferase units in a luminometer by first mixing the cell lysates (20 μl) with 100 μl of luciferase assay reagent to measure firefly luciferase activity and subsequently adding 100 μl of Renilla luciferase reagent to measure Renilla luciferase activity. The data were normalised to Renilla luciferase activity (internal control) and presented in arbitrary units. All experiments were performed in triplicate.

### Xenograft tumour model

A xenograft tumour model was established as our previous study.^28^ In brief, after the tumour size reached a mean diameter of 5 mm, the mice were treated with various regimens as follows: (a) Control group (*n* = 5, 0.9% saline, s.c.); (b) IFNα group (*n* = 5, 20,000 IU/day, s.c.). After 4 weeks, the mice were killed, and the tumour tissues were excised. The tissues were stained to detect the expression of indicated markers. This study was approved by the Ethics Committee of the Ninth People’s Hospital, Shanghai Jiao Tong University School of Medicine (Shanghai, China)

### Patient-derived tumour xenografts (PDXs)

PDXs are developed by surgically implanting tumour tissues directly from a patient into an immunocompromised mouse and are considered as the relative reliable xenograft models. The resulting tumours maintain the histologic characteristics of the primary tumour of the patient and mimic the response to chemotherapy in the clinic.^29^ Our HNSCC PDX model was established as previously described.^30^ The mice (three mice each group) were subjected to various regimens according to the scheme described for the xenograft tumour model. The tissues before and after IFNα treatment for 2 weeks were stained to detect the expression of indicated markers. This study was approved by the Ethics Committee of the Ninth People’s Hospital, Shanghai Jiao Tong University School of Medicine (Shanghai, China).

### Statistical analysis

Statistical analysis was performed with SPSS software 13.0 for Windows (SPSS Inc., USA). Excel and GraphPad Prism version 6 (GraphPad Software, San Diego, CA, USA) were employed to process initial data and for graph plotting. Student’s *t* test was performed to assess the statistical significance of differences. Survival analysis was conducted using the Kaplan–Meier method and log-rank test. The Cox proportional hazards model was used for univariate and multivariate analyses of disease prognosis. *P* *<* 0.05 is considered statistically significant. * indicates *P* *<* 0.05 and ** indicates *P* *<* 0.01. All values are expressed as the means ± standard deviation.

## Results

### Overexpression of IFNAR1 and constitutive activation of IFNα signalling are confirmed in HNSCC

**Fig. 1:**
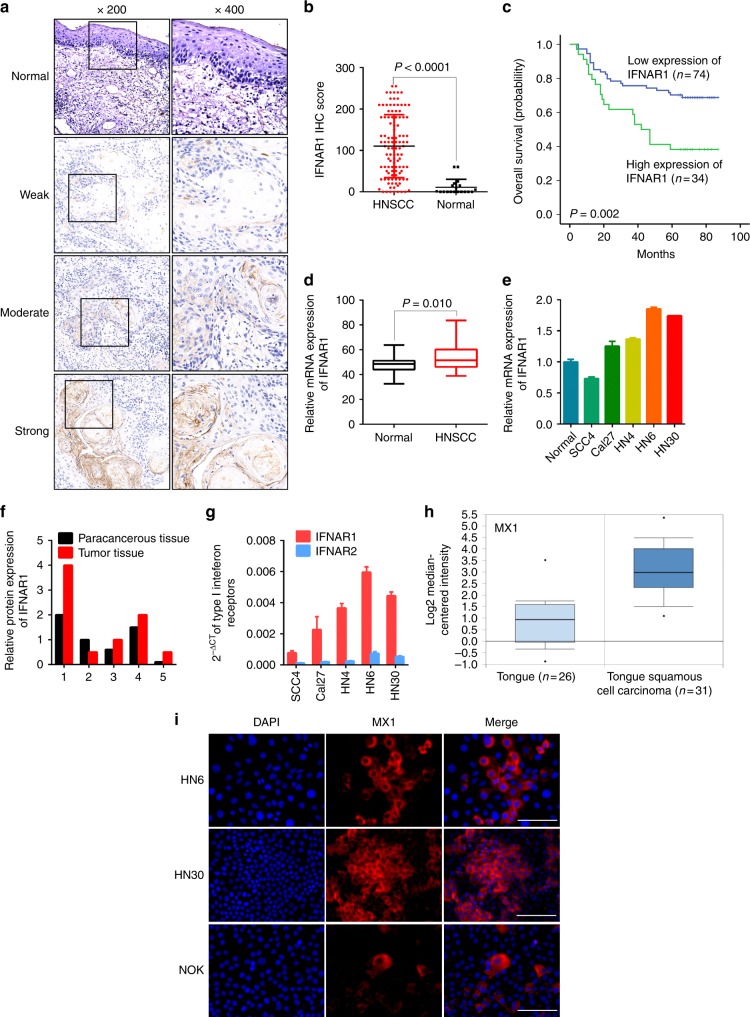
Overexpression of IFNAR1 in HNSCC patients and cell lines. **a** Representative images of IFNAR1 expression in HNSCC. **b** IHC score of IFNAR1 in HNSCCs (*n* = 108) and normal controls (*n* = 16). **c** Overall survival analysis based on IFNAR1 expression in 108 HNSCC patients. **d** Relative IFNAR1 mRNA expression in HNSCCs (*n* = 22) and the paired controls from the GEO database. **e** IFNAR1 mRNA expression in HNSCC cell lines and primary normal oral keratinocyte. **f** Relative IFNAR1 protein level in tumour (T) and paracancerous (P) tissues in five HNSCC patients. **g** mRNAs of type I interferon receptors was measured by real-time PCR in HNSCC cell lines. **h** *MX1* expression was analysed in tongue and tongue squamous cell carcinoma tissues in Oncomine website. **i** MX1 expression was detected in HN6, HN30 and NOK cells using immunofluorescence. Bar: 100 μm, magnification: ×200 and × 400, *: *P* *<* 0.05, **: *P* *<* 0.01. Data are expressed as mean ± S.D.

We first investigated the expression of IFNAR1 in 108 HNSCC patients and 16 normal control tissue samples using IHC. As shown in Fig. 1a, IFNAR1 was mainly located in the cellular membrane and was sometimes strongly expressed in the cytoplasm. The IHC score for IFNAR1 was significantly higher in HNSCC tissue samples than that in the control normal tissue samples (Fig. 1b, 110.4 ± 7.326 vs 11.11 ± 4.345, *P* *<* 0.0001). Moreover, IFNAR1 expression was significantly associated with TNM stage (*P* *=* 0.008) and pathologic differentiation (*P* *<* 0.001) in HNSCC patients (Supplementary Table. S1). The cutoff value of 150 for IFNAR1 expression was determined according to receiver operating characteristic curve (Supplementary Fig. S1). Patients with higher levels of IFNAR1 had poorer prognoses than that with lower levels of IFNAR1 (Fig. 1c, *P* *=* 0.002). Meanwhile, the mortality rate was 65.5% in the high IFNAR1 group and 55.9% in the low group of HNSCC patients from TCGA database (Supplementary Fig. S2). IFNAR1 expression and TNM stage were independent risk factors of prognosis (*P* *=* 0.013 and *P* *=* 0.011, respectively, Supplementary Table. S2). Meanwhile, *IFNAR1* expression was also higher in tumour tissues than that in adjacent normal tissues in the 22 HNSCC patients from the GEO database (Fig. 1d). The level of *IFNAR1* mRNA was also higher in 4/5 HNSCC cell lines than that in the control cell line (Fig. 1e), and the protein level of IFNAR1 was also increased in the tumour tissues compared with that in the adjacent normal tissues in 4/5 HNSCC patients (Fig. 1f, Supplementary Fig. S3). Furthermore, increased expression of *IFNAR1* in HNSCC patients and its association with worse prognosis were also confirmed in Oncomine (Supplementary Fig. S4A–D). The expression of IFNAR2 had a similar trend of upregulation in HNSCC cell lines, but its expression was low than IFNAR1 (Fig. 1g). MX1 is considered as an ideal and specific marker for the activity of the IFNα signalling pathway.^13^ Consistently, we observed an increase *MX1* (Fig. 1h) and *STAT1* expression in HNSCC patients in Oncomine (Supplementary Fig. S4E, F). There was positive correlation between *STAT1* and *MX1* mRNA in HNSCC (spearman coefficient: 0.75, Supplementary Fig. S5). Finally, immunofluorescence assay also confirmed the overexpression of MX1 in HN6 and HN30 cells compared with NOK cells (Fig. 1i). Together, although overexpression of IFNAR1 correlated with worse prognosis of HNSCC, activation of IFNα signalling was also evident as manifested by MX1 expression in tumour microenvironment of HNSCC. How the overexpression of IFNAR1 and constitutive activation of IFNα signalling promoted malignant phenotype in HNSCC need further study.

### High IFNAR1 expression correlates with immunosuppressive status in HNSCC patients

**Fig. 2:**
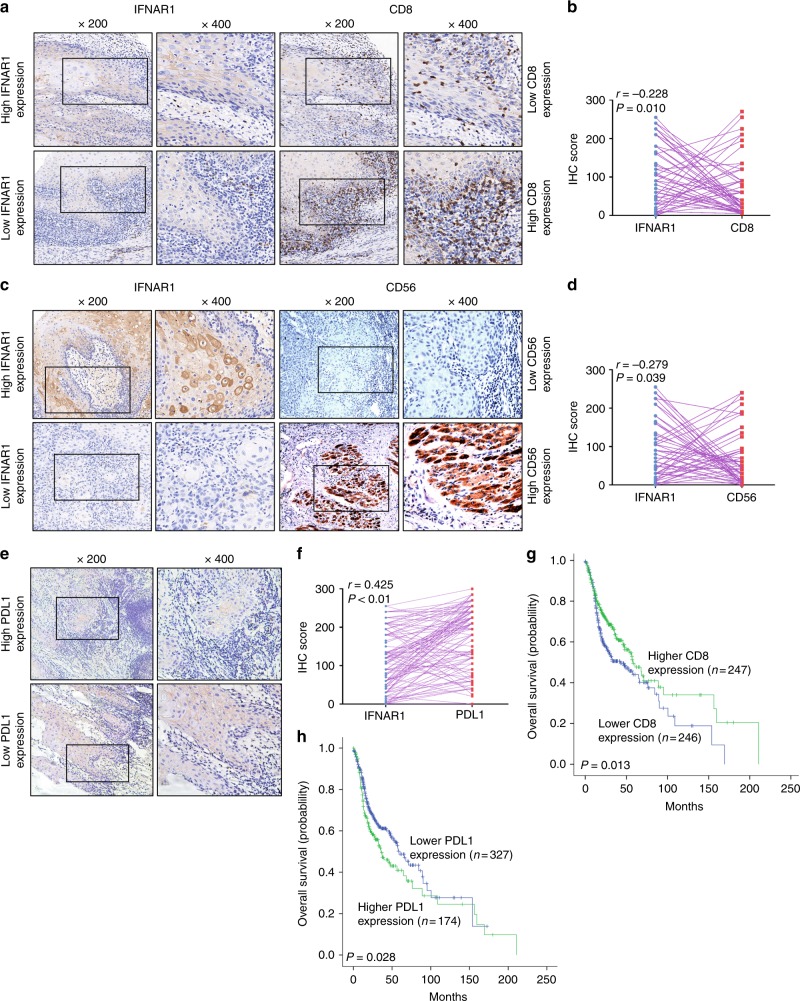
Higher IFNAR1 expression correlates with immunosuppressive status in HNSCC patients. **a** Representative images of IFNAR1 and CD8 immunohistochemistry (IHC) of 53 HNSCCs. **b** Correlation between IFNAR1 and CD8 IHC scores of 53 HNSCCs. Representative images of IFNAR1 and CD56 IHC **c** and their correlation of IHC score in 53 HNSCCs **d**. **e** Representative images of PDL1 IHC in 108 HNSCC patients. **f** Correlation between IFNAR1 and PDL1 IHC scores in 108 HNSCCs. **g** Kaplan–Meier analysis of overall survival of HNSCC patients (*n* = 493) in TCGA database with high versus low CD8 expression. **h** Overall survival of HNSCC patients (*n* = 501) in TCGA database with high versus low PDL1 expression. Magnification: × 200 and × 400, *: *P* *<* 0.05, **: *P* *<* 0.01

To explore why patients with higher IFNAR1 had poorer prognosis, the expression of immunosuppression-related molecules was detected in HNSCC patients. Tumours with high expression of IFNAR1 had few cytotoxic T lymphocytes (CTLs, CD8 + cells) and CD56^+^ NK infiltration, and vice versa (Figs. 2a, c). There was a negative correlation between IFNAR1 and CD8 expression (*r* *=* −0.228, *P* *=* 0.010, Fig. 2b) or between IFNAR1 and CD56 expression (*r* *=* −0.279, *P* *=* 0.039, Fig. 2d) in 53 HNSCC patients. In contrast, positive correlation between IFNAR1 and PDL1 expression was observed in the 108 HNSCC patients (*r* *=* 0.425, *P* *<* 0.010, Fig. 2e, f), and a frequent PDL1 gene amplification was also detected in several HNSCC subtype tumours from TCGA database (Supplementary Fig. S6). Moreover, there was positive correlation between *CD274* encoding PDL1 protein and *MX1* mRNA in TCGA HNSCC datasets (Supplementary Fig. S7). Furthermore, HNSCC patients with lower CD8 expression had poorer prognosis than that with higher CD8 expression (*P* *=* 0.013, Fig. 2g), whereas patients with low PDL1 expression had better prognosis than that with high PDL1 expression (*P* *=* 0.028, Fig. 2h). Therefore, the poor prognosis of HNSCC patients with high IFNAR1 expression may be attributed to the strong immunosuppressive status (i.e., low CD8^+^ T and CD56^+^ NK expression, high PDL1 expression) of the tumour microenvironment.

### IFNα promotes the expression of PDL1 through IFNAR1/STAT1 signalling in HNSCC cells

**Fig. 3:**
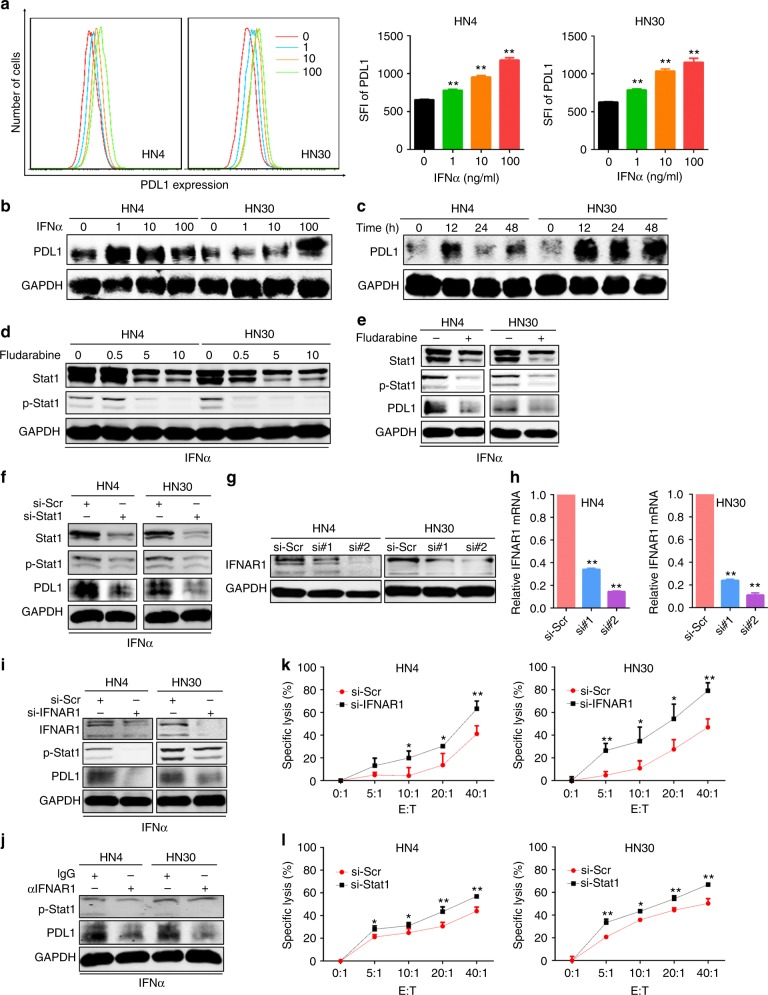
IFNα promotes PDL1 expression through IFNAR1/STAT1 signalling in HNSCC cells. **a** Cell surface PDL1 expression in HN4 and HN30 cells was analysed by flow cytometry after 0, 1, 10 and 100 ng/ml IFNα treatment for 48 h. **b** Western blot of PDL1 expression in HN4 and HN30 cells after 0, 1, 10 and 100 ng/ml IFNα treatment for 48 h. **c** PDL1 expression was detected in HN4 and HN30 cells by western blot under 100 ng/ml IFNα treatment as indicated time. **d** P-Stat1 (Tyr701) and Stat1 levels were determined in HN4 and HN30 cells at 12 h after 0, 0.5, 5 and 10 μm fludarabine (a specific Stat1 inhibitor) treatment. **e** In response to 100 ng/ml IFNα treatment, p-Stat1, Stat1 and PDL1 levels were analysed with or without 10 μm fludarabine treatment for 12 h. **f** Western blot of Stat1, p-Stat1 and PDL1 was performed after STAT1 siRNA transfection for 24 h and then stimulation with 100 ng/ml IFNα for 24 h. Efficiency for IFNAR1 gene silencing was confirmed by western blot **g** and real-time PCR **h**. **i** After transfection with siRNAs against IFNAR1 for 24 h, IFNAR1, p-Stat1 and PDL1 levels were detected in response to 100 ng/ml IFNα treatment for 24 h. (**J**) HN4 and HN30 cells were pre-treated with IFNAR1-blocking antibody (10 μg/ml) or normal IgG antibody for 4 h and then incubated with 100 ng/ml IFNα for 12 h. P-Stat1 and PDL1 levels were detected. **k**, **l** Cell viability luciferase assays after HN4 and HN30 cells transfected with siRNAs against STAT1 **k** or IFNAR1 **l** for 48 h, and then seeded at the density of 1 × 10^3^ cells per well in a 96-well plate and incubated with NK cells for 4 h at various effector/target (E:T) cell ratios as indicated. *: *P* *<* 0.05, **: *P* *<* 0.01. Data are expressed as mean ± S.D.

We then explored whether IFNα can promote the expression of PDL1 in HNSCC. The surface expression and total expression of PDL1 were induced by IFNα treatment in HN4 and HN30 cells, especially at 12 h with 100 ng/ml IFNα (Fig. 3a–c). To explore whether the phosphorylation of Stat1 plays a critical role in IFNα-induced PDL1 expression, we used fludarabine (a specific inhibitor of p-Stat1^31^) and our results showed that it not only inhibited the activation of Stat1 in a dose- and time-dependent manner (Fig. 3d, Supplementary Fig. S8), but also significantly attenuated IFNα-induced PDL1 expression in HN4 and HN30 cells (Fig. 3e). Similarly, silencing of Stat1 using siRNAs also decreased PDL1 expression in response to IFNα treatment (Fig. 3f). Moreover, although siRNAs against IFNAR1 decreased IFNAR1 expression (Fig. 3g, h), they also significantly inhibited PDL1 expression in response to IFNα stimulation in HN4 and HN30 cells (Fig. 3i). Consistent with this, an IFNAR1-blocking antibody also inhibited PDL1 expression (Fig. 3j). Furthermore, besides IFNα, IFNβ and IFNγ can also induced PDL1 expression in HNSCC cells (Supplementary Fig. S9). Finally, we observed that HN4 and HN30 cells were more susceptible to NK cell-mediated immune cell lysis upon *STAT1* or *IFNAR1* gene silencing (Fig. 3k, l). These results taken together strongly suggested that IFNα promoted expression of PDL1 through IFNAR1/Stat1 signalling in HNSCC cells.

### IFNα promotes the expression of PDL1 in tumour cells and PD1 in immune cells

**Fig. 4:**
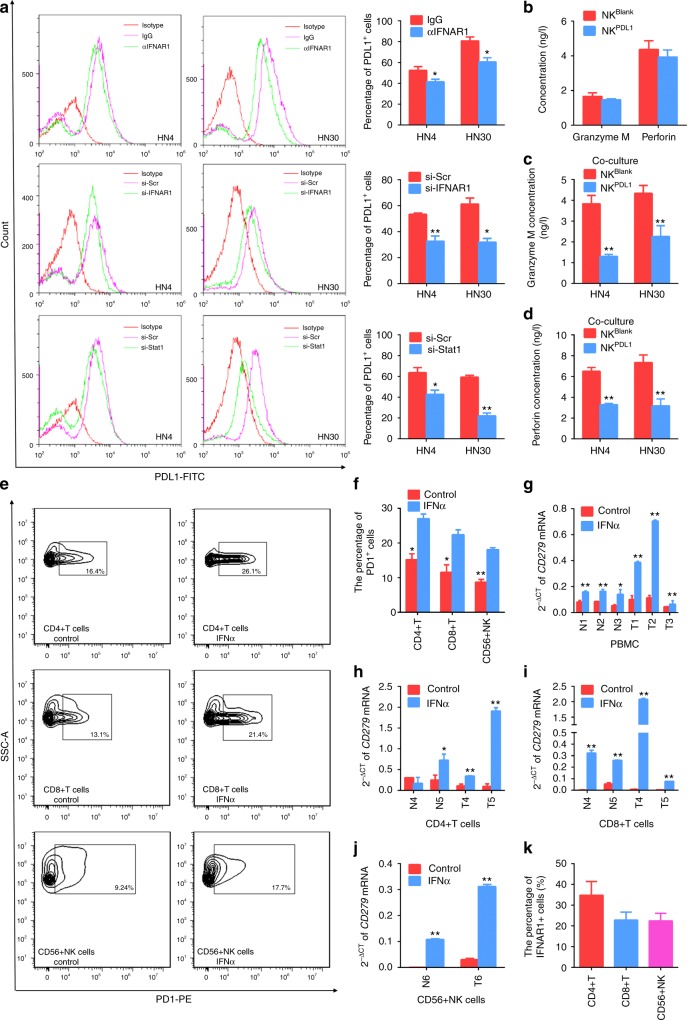
IFNα promotes the expression of PDL1 in HNSCC cells and PD1 in immune cells. **a** HN4 and HN30 cells were pre-treated with IFNAR1-blocking antibody (10 μg/ml) or normal IgG antibody for 4 h and then incubated with 100 ng/ml IFNα for 24 h. After transfection with IFNAR1 and Stat1-specific siRNAs for 24 h, PDL1 levels were detected in response to 100 ng/ml IFNα treatment for 24 h. The surface PDL1 expression was detected using flow cytometry. **b**–**d** ELISA assays of Granzyme M and perforin released by NK cells (normal and treated with 10 μg/ml recombinant PDL1 protein for 24 h) co-cultured with or without HN4 and HN30 cells. **e** Flow cytometry analyses of the surface PD1 expression of CD4^+^ T, CD8^+^ T and CD56^+^ NK cells isolated from PBMC of HNSCC patients and treated with 0 and 100 ng/ml IFNα for 24 h. **f** The percentage of PD1^+^ cells among immune cells after 100 ng/ml IFNα incubation for 24 h was analysed by flow cytometry. **g** Real-time PCR analysis of *CD279* mRNA in IFNα-treated (100 ng/ml, 12 h) PBMCs isolated from three healthy controls and three HNSCC patients. **h**–**i** *CD279* mRNA expression in IFNα-treated (100 ng/ml, 12 h) CD4^+^ T **h** and CD8^+^ T **i** cells from two healthy controls and two HNSCC patients. **j** *CD279* mRNA expression in IFNα-treated (100 ng/ml, 12 h) CD56^+^ NK cells from one healthy control and one HNSCC patient. **k** The surface IFNAR1 expression on immune cells from three HNSCC patients was analysed by flow cytometry. *: *P* *<* 0.05, **: *P* *<* 0.01. Data are expressed as mean ± S.D.

Moreover, the percentage of PDL1^+^ cells were significantly inhibited after neutralising antibody of IFNAR1 incubation and siRNAs for IFNAR1 and Stat1 treatment (Fig. 4a). To investigate whether PDL1 expression had impacts on NK cells, we tested recombinant human PDL1 protein in NK cells culture. As shown in Fig. 4b–d, treatment with PDL1 protein alone did not affect the release of Granzme M and perforin of NK cells, but PDL1 treatment in the presence of the tumour cells (i.e., HN30 and HN4) under the co-culture condition inhibited the release of Granzme M and perforin from NK cells. Granzyme M was constitutively highly expressed in NK cells as was perforin.^32^ This result indicated that increased expression of PDL1 in tumour microenvironment could attenuate the killing activation of NK cells.

In addition to the tumour cells, we also explored the effect of IFNα stimulation on immune cells in HNSCC patients. We observed that the expression of surface PD1 (the receptor of PDL1) was significantly elevated in CD4^+^ T cells, CD8^+^ T cells and CD56^+^ NK cells (Fig. 4e, f). In addition, our results showed that *CD279* mRNA encoding PD1 was elevated in PBMCs from healthy controls and HNSCC patients, but the elevation was more prominent in PBMCs from HNSCC patients treated with IFNα (Fig. 4g). Moreover, *CD279* mRNA level increased in CD4^+^ T cells in samples from all but one healthy control (Fig. 4h), and in CD8^+^ T cells and NK cells from both healthy controls and HNSCC patients in response to IFNα treatment (Fig. 4i, j). In addition, the surface IFNAR1 was also widely expressed on CD4^+^ T, CD8^+^ T and CD56^+^ NK cells (Fig. 4k, Supplementary Fig. S10), and there was a positive correlation between *STAT1* and *PDCD1* mRNA encoding PD1 protein in HNSCC (Supplementary Fig. S11). These results suggested that IFNα could promote formation of an immunosuppressive microenvironment by increasing the expression of PDL1 in tumour cells and PD1 in immune cells of HNSCC.

### IFNα transcriptionally activates the expression of PDL1 through p-Stat1 in HNSCC cells

**Fig. 5:**
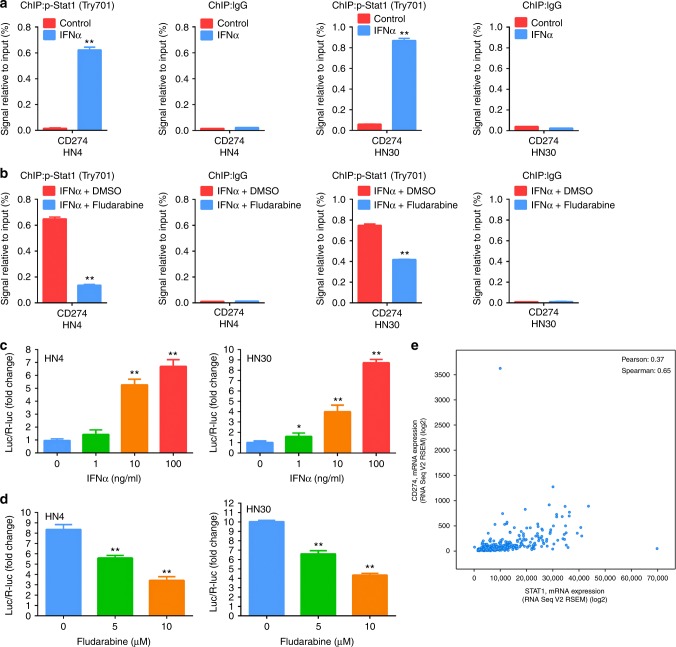
IFNα transcriptionally activates PDL1 expression in HNSCC. **a** HN4 and HN30 cells were incubated with 100 ng/ml IFNα for 48 h. ChIP assay was performed with anti-p-Stat1 (Tyr701) and control rabbit IgG antibodies. **b** HN4 and HN30 cells were incubated with 100 ng/ml IFNα with 10 μm fludarabine or DMSO for 24 h. ChIP assay was performed with anti-p-Stat1 (Tyr701) and control rabbit IgG antibodies. **c** *CD274* promoter activity was detected in HN4 and HN30 cells at 24 h after stimulation with IFNα. **d** *CD274* promoter activity was detected in HN4 and HN30 cells under incubation with 100 ng/ml IFNα and indicated fludarabine for 24 h. **e** Correlation between *STAT1* and *CD274* mRNA levels in HNSCC patients was analysed using TCGA database. *: *P* *<* 0.05, **: *P* *<* 0.01. Data are expressed as mean ± S.D.

As Stat1 is the main transcriptional factor in interferon signalling, we hypothesise that IFNα promotes PDL1 expression through Stat1-mediated transcription activation. In support of this, our ChIP assay showed that p-Stat1 (Tyr701) bound to the upstream promoter region of the *CD274* gene in IFNα-stimulated HN4 and HN30 cells (Fig. 5a), but this binding could be inhibited by fludarabine (Fig. 5b). To further confirm whether IFNα can activate *CD274* transcription, we assessed the transcriptional activation of the *CD274* promoter by IFNα using the dual-luciferase reporter system. As shown in Fig. 5c, d, the activity of the *CD274* promoter was significantly activated by IFNα stimulation, but inhibited by fludarabine, in a dose-dependent manner. Finally, we also observed a positive correlation between *STAT1* and *CD274* mRNA in HNSCC patients from TCGA database (Pearson’s correlation coefficient: 0.37, Spearman’s correlation coefficient: 0.65, Fig. 5e). These results indicated that IFNα could induce the transcriptional expression of PDL1 through Stat1 activation in HNSCC cells.

### IFNα promotes the expression of PDL1/PD1 in both the xenograft and PDXs model

**Fig. 6:**
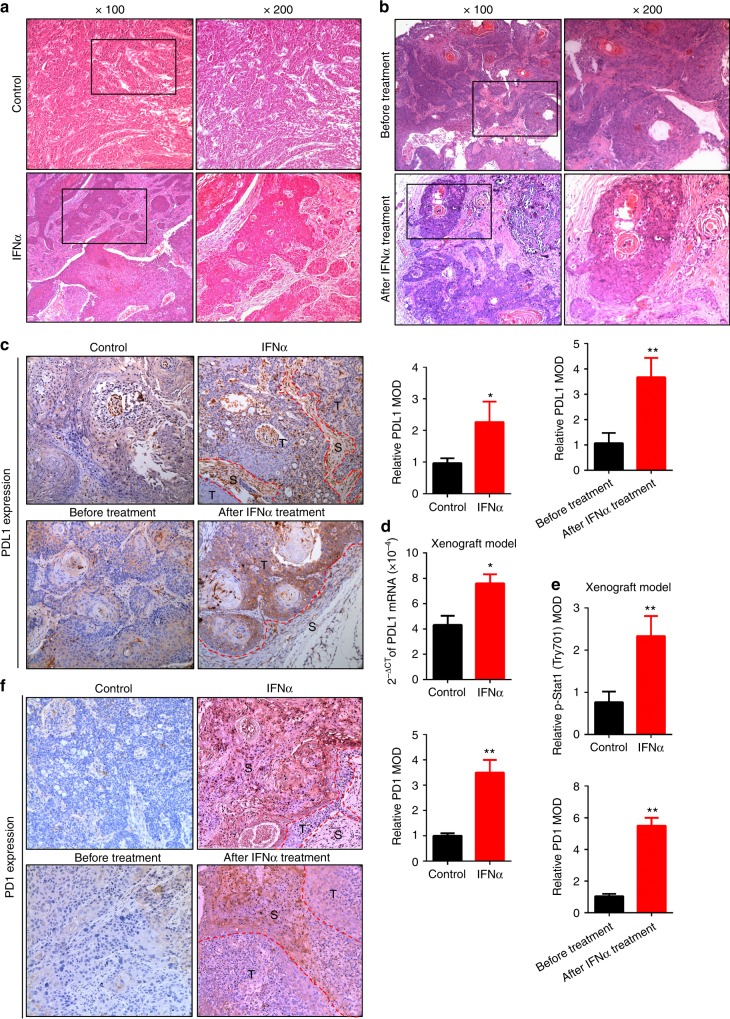
IFNα promoted PDL1 expression in vivo. **a**, **b** Representative images of tumour H&E staining from xenograft **a** and PDX **b** mice treated with or without IFNα (20,000 IU/day, s.c) for 2 weeks. **c** Representative images of IHC of PDL1 expression from xenograft and PDX mouse models. **d** PDL1 mRNA expression was quantified by real-time PCR using fresh xenograft tumour samples. **e** Relative IHC quantitative analysis of p-Stat1 (Tyr701) expression in xenograft tumours. **f** Representative images of IHC of PD1 expression in tumours from xenograft and PDX mouse models. Magnification: × 200, T: tumour, S: stroma, *: *P* *<* 0.05, **: *P* *<* 0.01. Data are expressed as mean ± S.D.

**Fig. 7:**
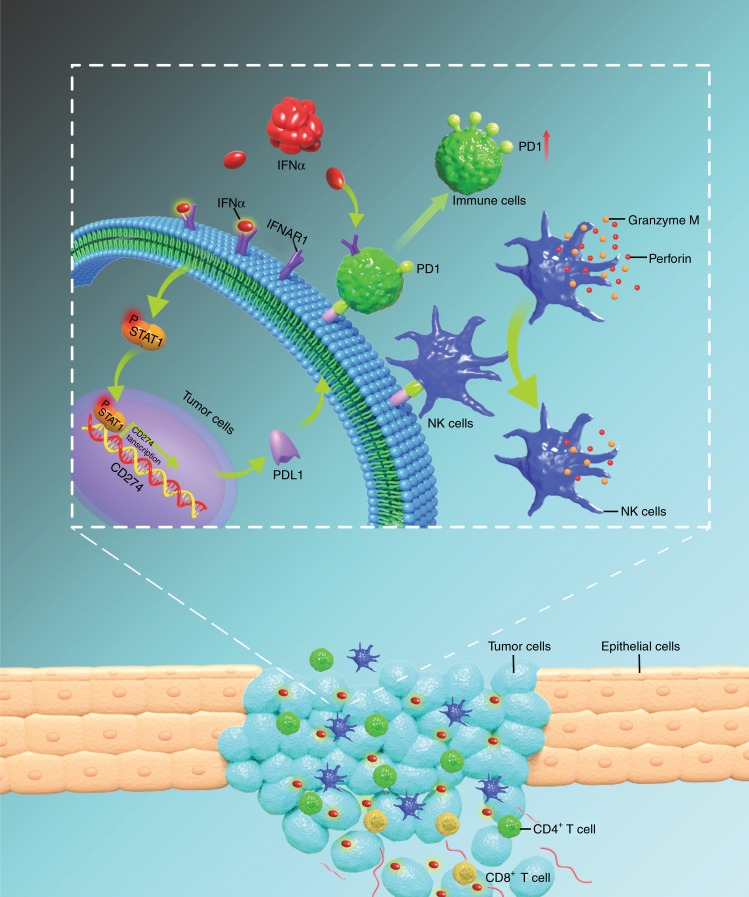
The schematic diagram of the mechanisms shows that endogenous IFNα promotes formation of immunosuppressive tumour microenvironment through upregulation of PDL1 and PD1 on the surfaces of tumour cells and immune cells, respectively, in HNSCCs. Increased expression of PDL1 and PD1 inhibits the killing activity of immune cells

We further used both xenograft tumour and PDXs models to validate our in vitro results. We found that the composition of tumours in the xenograft model was relatively uniform with low heterogeneity (Fig. 6a), whereas tumours from PDXs models were more heterogeneous with many histological and genetic features of the primary tumours (Fig. 6b) as observed in many other PDX tumour models.^33^ Increased stromal cell numbers and necrotic elements were observed in response to IFNα treatment in vivo. Moreover, PDL1 expression was markedly elevated after IFNα treatment in two models (Fig. 6c). PDL1 was mainly expressed in the nuclei or cytoplasm in the xenograft model, whereas it was expressed on the cell membrane in the PDX model. The differential expression patterns may be attributable to the differences in the tumour microenvironment between the two models. In addition, PDL1 mRNA, p-Stat1 and PD1 expression were elevated after IFNα treatment in the xenograft tumours (Fig. 6d–f, Supplementary Fig. S12). These results further supported that endogenous IFNα promoted the immunosuppressive status in tumour microenvironment by increasing PD1 expression in immune cells and PDL1 expression in tumour cells through IFNAR1/Stat1 signalling (Fig. 7).

## Discussion

Elucidation of the mechanisms by which IFNα promotes the expression of PDL1 that contributes to immunosuppression may improve the efficacy of ICB in HNSCC. Here, we showed that IFNAR1 is an independent predictor of survival and that the overexpression of IFNAR1 and the constitutive activation of IFNα signalling are correlated with the immunosuppression in HNSCC. Moreover, our results demonstrated that endogenous IFNα can promote the expression of PDL1 in HNSCC cells and PD1 in immune cells, and that the combination of PD1 and PDL1 suppresses the killing activity of NK cells. Consistent with this, knocking down IFNα signalling could enhance the cytotoxic effect of NK cells on HNSCC cells. Thus, our results together strongly support that IFNα plays an important role in immunosuppression in HNSCC.

The therapeutic blockade of the PD1/PDL1 pathway results in significant tumour responses in a specific subset of patients, but resistance is also common. It has been reported that persistent interferon signalling orchestrates PDL1-dependent and PDL1-independent resistance to ICB.^34^ So, for most studies have been focused on IFNγ-induced PDL1 expression,^35,36^ but there were distinct biological functions between IFNγ and IFNα in tumour immunology.^37^ Therefore, our study is of great significance since it is the first to demonstrate that IFNα can strongly promote the transcriptional expression of PDL1 and the formation of an immunosuppressive microenvironment through IFNAR1 in HNSCC.

IFNAR1’s role in tumours appears to be complex. As the patients enroled in our study did not have a history of interferon therapy, our results that the patients with higher IFNAR1 expression had poorer prognosis suggested that overexpression of IFNAR1 can promote the progression of HNSCC. In contrast to this, the lack of IFNAR1 expression was shown to predispose mouse embryonic fibroblasts to cellular transformation.^38^ It was also shown that the metastatic dissemination of breast cancer is accelerated in IFNAR1^−/−^ mice, as well as in mice depleted of NK cells and T cells,^39^ and that IFNAR1 is downregulated in colorectal cancer and promotes the generation of immune-privileged niches.^40^ The differences in tumour origination, micro-ecological environment, physiological function, and genetic heterogeneity et al may explain the opposite function of IFNAR1 between colorectal cancer and HNSCC. These studies suggested that IFNAR1 acted as a tumour suppressor in those cancers. Similar to our observations, it has also been reported that higher proportions of cells with IFNAR1 mRNA expression were detected in colorectal tumours than that in normal tissues^41^ and that 91.5% of pancreatic tumours and 88.9% of the periampullary tumours expressed IFNAR1, among which 23.4 and 13.0%, respectively, were strongly positive.^42^ The inconsistent results indicated that the function of IFNAR1 may vary in different solid tumours.

Although both IFNAR1 and IFNAR2 are receptors for type I interferons, intracellular signalling cascades are only activated by IFNAR1.^43^ Because of its central role in IFNα signalling and relative high expression compared to IFNAR2, IFNAR1 was mainly investigated in our study. Our results confirmed that endogenous IFNα secretion and IFNα signalling were constitutively active under the physical condition as manifested by a high level of MX1, Stat1 and IFNAR1 expression in HNSCC cells. As IFNα can be secreted by all the nucleated cells, our results of the correlation of high IFNAR1 expression with an immunosuppressive tumour microenvironment (low CD8, CD56 and high PDL1 expression), may help to explain why high IFNAR1 expression is an independent indicator of poor prognosis of HNSCC. An immunosuppressive tumour microenvironment leads to CTL exhaustion, NK cell apoptosis, which promotes immune escape and contributes to aggressive progression.^44^

Immune dysfunction in HNSCC has been extensively reported.^3^ The ICB that blocks cytotoxic T lymphocyte-associated protein 4 (e.g., ipilimumab) or PD1 (e.g., nivolumab and pembrolizumab) release the inhibitory cues imposed by tumour-derived signals and enable T cells to resume their immune activities.^45^ However, only a small proportion of patients (~ 15%) with R/M-HNSCC achieved durable remissions and prolonged survival.^46^ Thus, it is unclear why most of the patients do not respond to PD1/PDL1-targeted therapies. In glioma cells, the presence of constitutive interferon beta promoted immune escape by upregulating PDL1.^13^ Here, we show that endogenous IFNα signalling is constitutively active in HNSCC, and that IFNα can promote PDL1 expression through IFNAR1/Stat1 signalling in HNSCC. Given that activation of IFNα signalling with high PDL1 expression under physiological condition may impede the antitumour effect of PD1/PDL1-targeted therapies, identification of an IFNα activation signature prior to the use of ICB may help to identify the patients who can respond to the treatment.^34^ For those patients with a strong IFNα signature, it might be beneficial to initially block IFNα signalling to make the patients more responsive to ICB.

IFNα is a double-edged sword in cancer, as it not only provides the necessary inflammatory signals but also initiates a feedback suppression of both immune and cancer cells.^47^ Traditionally, IFNα is considered beneficial and necessary to both promote T-cell responses and prevent metastases.^47^ However, accumulating evidences indicate that IFNα can also promote immunosuppression in several cancers. For instance, IFNα directly promotes PD1 transcription and limits the duration of T cell-mediated immunity in antigen-specific CD8 + T cells.^48^ Moreover, PDL1 was upregulated by IFNα in BRAF-mutant melanoma cells.^49^ Our results of upregulation of PDL1 in HNSCC cells and of PD1 in immune cells after IFNα treatment provided us great insights into the ICB resistance associated with anti-PD1/PDL1 treatment failure in HNSCCs. In addition, consistent with the emerging concept that blocking IFNα signalling may help to restore immune surveillance and enhance the effect of ICB.^50,51^ Our results showed that increasing PDL1 expression in tumour cells suppresses the release of granzyme M and perforin by NK cells to kill tumour cells, and that attenuating the IFNAR1/Stat1 signalling could enhance the NK cell-mediated cytotoxicity against HNSCC cells. Finally, our study also demonstrated that IFNα can increase the surface PD1 expression in CD4^+^ T, CD8^+^ T and CD56^+^ NK cells. Taken together, all these results indicated that IFNα-induced PDL1 and PD1 expression may inhibit antitumour immunity and tumour suppression by ICB, and that blocking IFNα signalling could be an effective strategy to enhance the therapeutic efficacy of ICB in HNSCCs.

In summary, we demonstrate that endogenous IFNα promotes the expression of PDL1 through IFNAR1/Stat1 in tumour cells and PD1 in immune cells, which contributes to immunosuppression formation in HNSCCs. Blocking IFNα signalling can revert the immunosuppressive tumour microenvironment and thus enhance the effect of ICBs.

## Electronic supplementary material

Supplementary Tables and Figures
