# Supplementary material for: Interferon-alpha promotes immunosuppression through IFNAR1/STAT1 signalling in head and neck squamous cell carcinoma
Source: Br J Cancer. 2018 Dec 17;120(3):317–30. doi: 10.1038/s41416-018-0352-y (PMC6353953; doi:10.1038/s41416-018-0352-y)

**Supplementary Table S1** Demographic characteristic of the patients by IFNAR1 expression

| Characteristics | Patients (%) | IFNAR1 score | | parametric test value | *P* value |
| --- | --- | --- | --- | --- | --- |
|  |  | Mean | SEM |  |  |
| Gender | | | | | |
| Male | 71(65.7) | 112.4 | 12.8 | *t*=0.198 | 0.844 |
| Female | 37(34.3) | 109.3 | 9.0 |  |  |
| Age | | | | | |
| <60 years | 52(48.2) | 103.4 | 11.0 | *t*=-0.909 | 0.365 |
| ≥60 years | 56(51.8) | 116.8 | 9.8 |  |  |
| Site | | | | | |
| Tongue | 45(41.7) | 98.7 | 10.0 | *F*=0.889 | 0.473 |
| Buccal | 22(20.4) | 120.5 | 17.2 |  |  |
| Gum | 10(9.3) | 144.0 | 24.2 |  |  |
| Floor of mouth | 10(9.3) | 101.0 | 17.4 |  |  |
| others | 21(19.3) | 113.1 | 17.4 |  |  |
| TNM stage | | | | | |
| I/II | 62(57.4) | 93.9 | 9.2 | *t*=-2.691 | **0.008** |
| III/IV | 46(42.6) | 132.6 | 11.2 |  |  |
| Pathologic differentiation | | | | | |
| Well | 43(39.8) | 68.5 | 8.3 | *t*=-5.176 | **.000** |
| Moderately/poorly | 65(60.2) | 138.1 | 9.4 |  |  |
| Smoking status^a^ | | | | | |
| Yes | 68(63.0) | 105.9 | 9.2 | *t=*-0.788 | 0.433 |
| No | 40(37.0) | 117.9 | 12.2 |  |  |
| Alcohol use^b^ | | | | | |
| Yes | 77(71.3) | 104.6 | 8.6 | *t*=-1.246 | 0.216 |
| No | 31(28.7) | 124.7 | 13.9 |  |  |

a. Former/current smokers defined as at least a one pack-year history of smoking.
b. Positive alcohol use was defined as current alcohol use of more than one drink per day for 1 year (12 ounces of beer with 5% alcohol, or 5 ounces of wine with 12–15% alcohol, or one ounce of liquor with 45–60% alcohol).All other patients were classified as negative alcohol use.

| **Supplementary Table S2**. Univariate and multivariate cox regression models for estimating the overall survival | | | |
| --- | --- | --- | --- |
| **Characteristic** | **HR** | **95%CI** | ***P* value** |
| **Univariate analysis** | | | |
| Overall survival | | | |
| Age (<60 y vs ≥ 60 y) | 1.389 | 0.761~2.535 | 0.284 |
| Gender (male vs female) | 0.891 | 0.473~1.681 | 0.722 |
| Alcohol history( Non-drinkers vs drinker ) | 0.928 | 0.478~1.802 | 0.825 |
| Smoking history( nonsmoker vs smoker ) | 0.870 | 0.465~1.617 | 0.653 |
| Pathological differentiation（ⅠvsⅡ/Ⅲ） | 2.754 | 1.359~5.581 | **0.005** |
| TNM stage (Ⅰ/Ⅱvs Ⅲ/Ⅳ) | 2.483 | 1.359~4.536 | **0.003** |
| IFNAR1 expression (low vs high) | 2.457 | 1.357~4.449 | **0.003** |
| **Multivariate analysis** | | | |
| Overall survival | | | |
| IFNAR1 expression (low vs high) | 2.153 | 1.178~3.933 | **0.013** |
| TNM stage (Ⅰ/Ⅱvs Ⅲ/Ⅳ) | 2.205 | 1.196~4.067 | **0.011** |

**Supplementary Figure Legends**

**Supplementary Figure S1.** Receiver operating characteristic (ROC) curve was determined according to IFNAR1 IHC score.

**Supplementary Figure S2.** Overall survival of HNSCC patients was analyzed according to IFNAR1 expression in TCGA database.

**Supplementary Figure S3.** IFNAR1 protein expression was detected in tumor (T) and paracancerous (P) tissues in five HNSCC patients using western blot.

**Supplementary Figure S4.** IFNAR1, MX1 and STAT1 expression in HNSCC patient tissues from Oncomine. (A) IFNAR1 expression in tongue squamous cell carcinoma patients (1, n=26) and in controls (0, n=12). (B) IFNAR1 expression in oral cavity squamous cell carcinoma patients (1, n=57) and in controls (0, n=22). (C) IFNAR1 expression in HNSCC patients who had died within 5 years (2, n=15) and in patients who were alive at 5 years (1, n=14) and in controls (0, n=9). (D) IFNAR1 expression in HNSCC patients who had died within 5 years (2, n=105) and in patients who were alive at 5 years (1, n=26) and in controls (0, n=497). (E) STAT1 expression was higher in oral cavity squamous cell carcinoma patients (1, n=16) than in controls (0, n=4). (F) STAT1 expression in HNSCC patients (1, n=41) and in controls (0, n=13).

**Supplementary Figure S5.** The correlation between *STAT1* and *MX1* mRNA was analyzed in 279 HNSCC patients for TCGA database.

**Supplementary Figure S6** Frequency of PDL1 alteration was analyzed in HNSCC datasets from TCGA database.

**Supplementary Figure S7.** The correlation between *CD274* and *MX1* mRNA was analyzed in 488 HNSCC patients for TCGA, PanCancer Atlas.

**Supplementary Figure S8.** Phospho-Stat1 (Tyr701) expression was detected by western blot in HN4 and HN30 cells after 10 μM fludarabine treatment for 0, 6, 12, and 24 h.

**Supplementary Figure S9.** STAT1, STAT3 and PDL1 expression were detected in HN4 and HN30 cells after 200 ng/ml IFNα, IFNβ and IFNγ stimulation for 24 h.

**Supplementary Figure S10.** The surface IFNAR1 expression on CD4^+^ T cell，CD8^+^ T cell and CD56^+^ NK cell was analyzed by flow cytometry.

**Supplementary Figure S11.** The correlation between *STAT1* and *PDCD1* mRNA encoding PD1 protein was analyzed in 502 HNSCC patients for TCGA database.

**Supplementary Figure S12.** Activation of Stat1 (Tyr701) was detected in xenograft tumors. Magnification: 200×.

Supplementary Figure S1


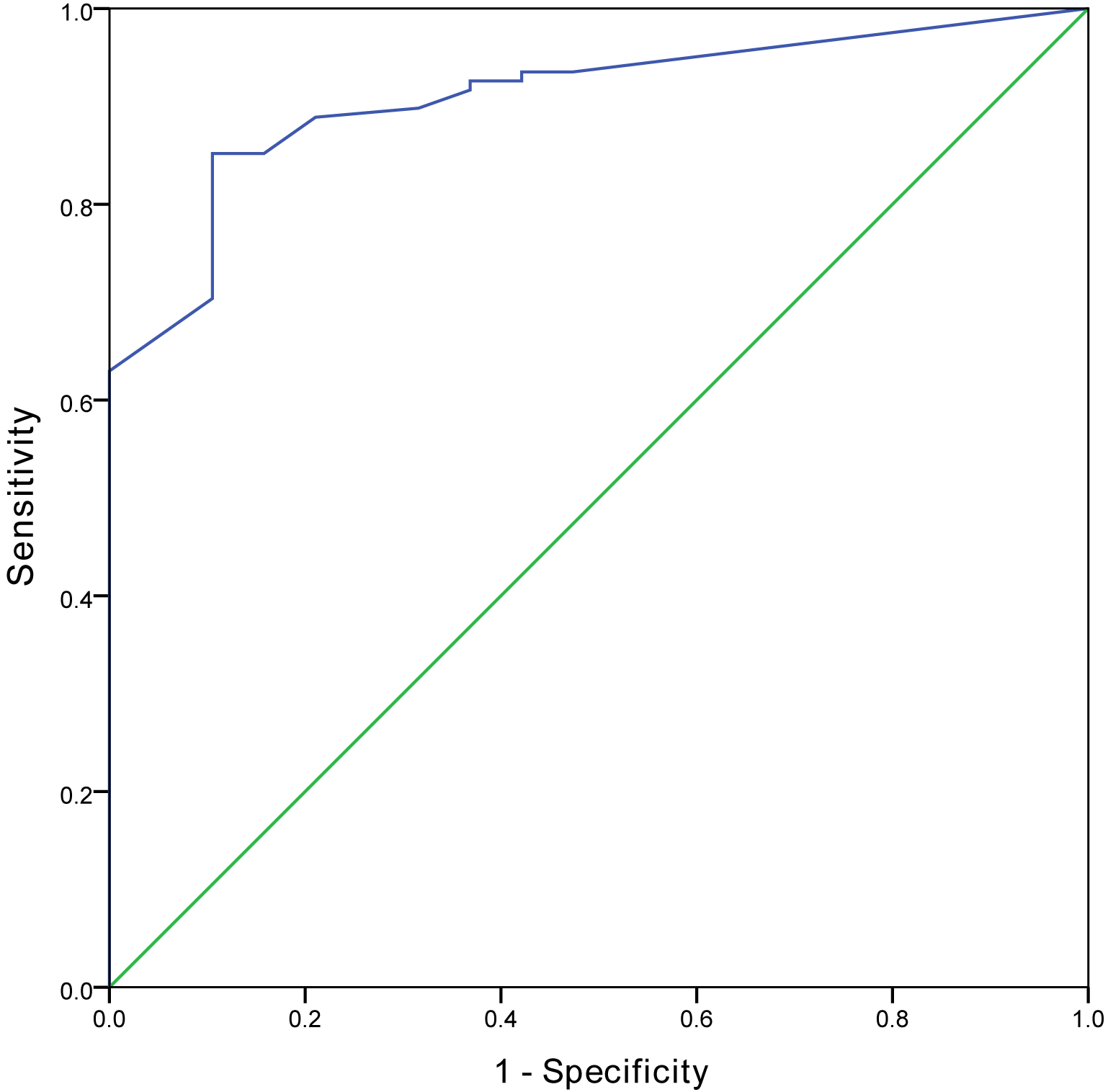


Supplementary Figure S2


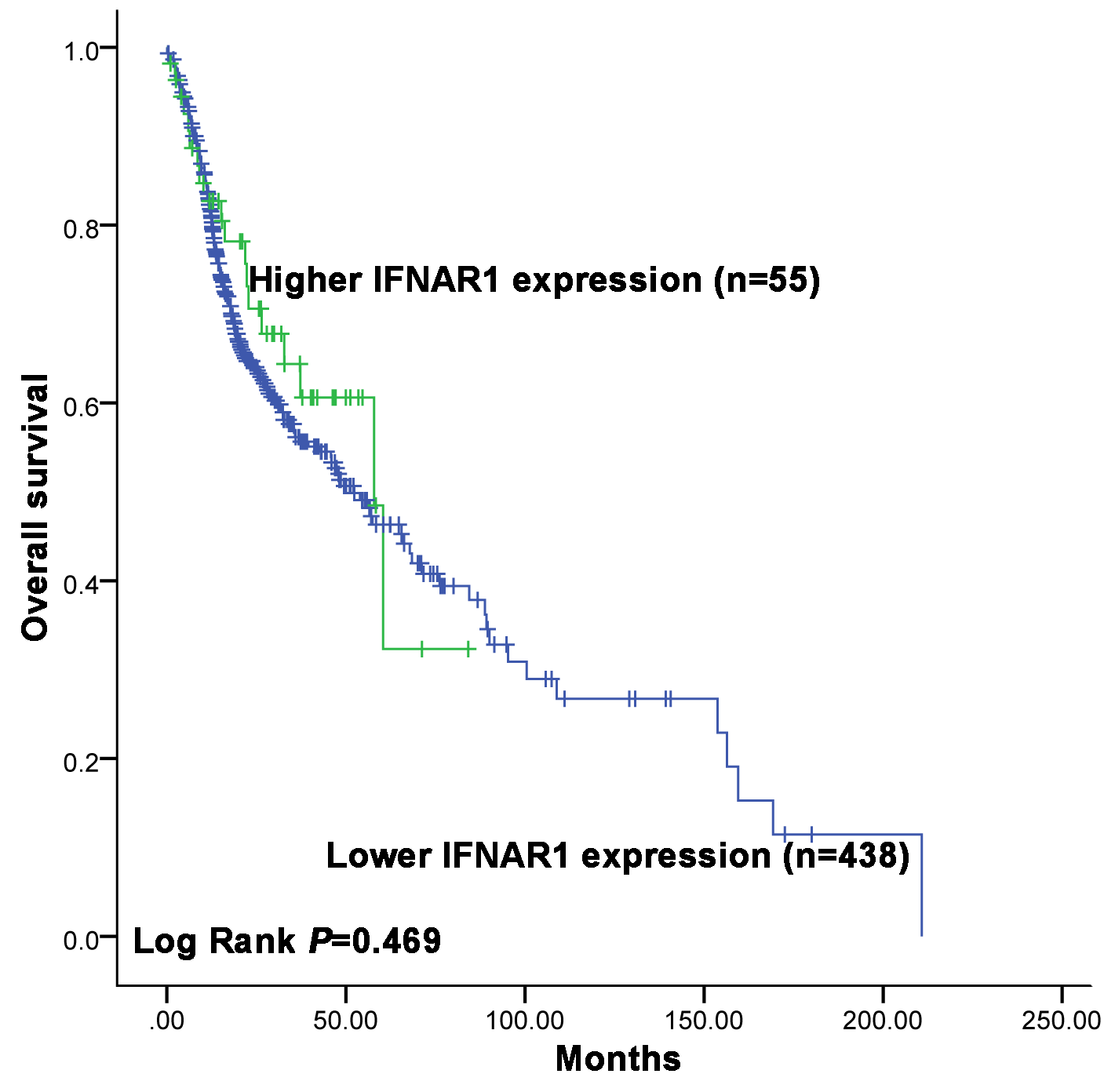


Supplementary Figure S3


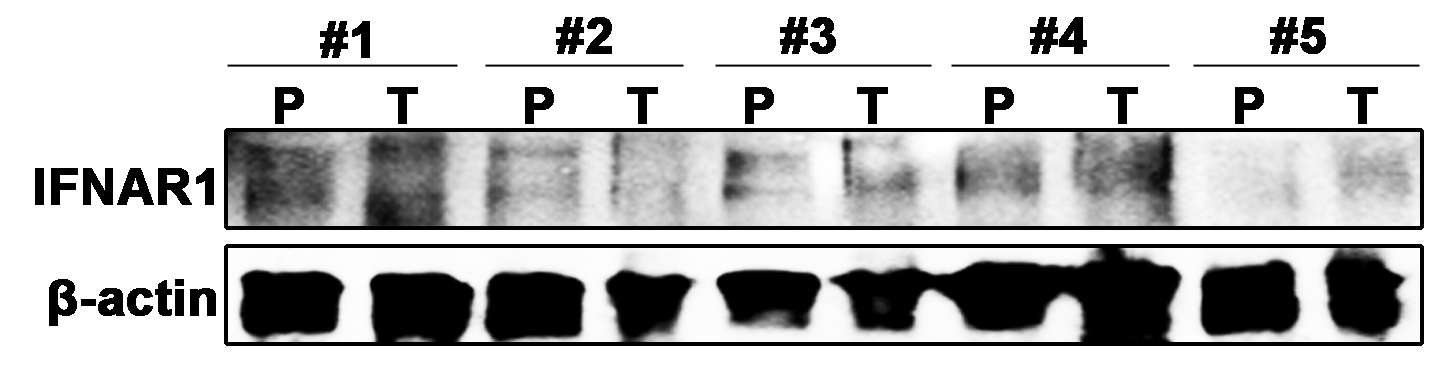


Supplementary Figure S4


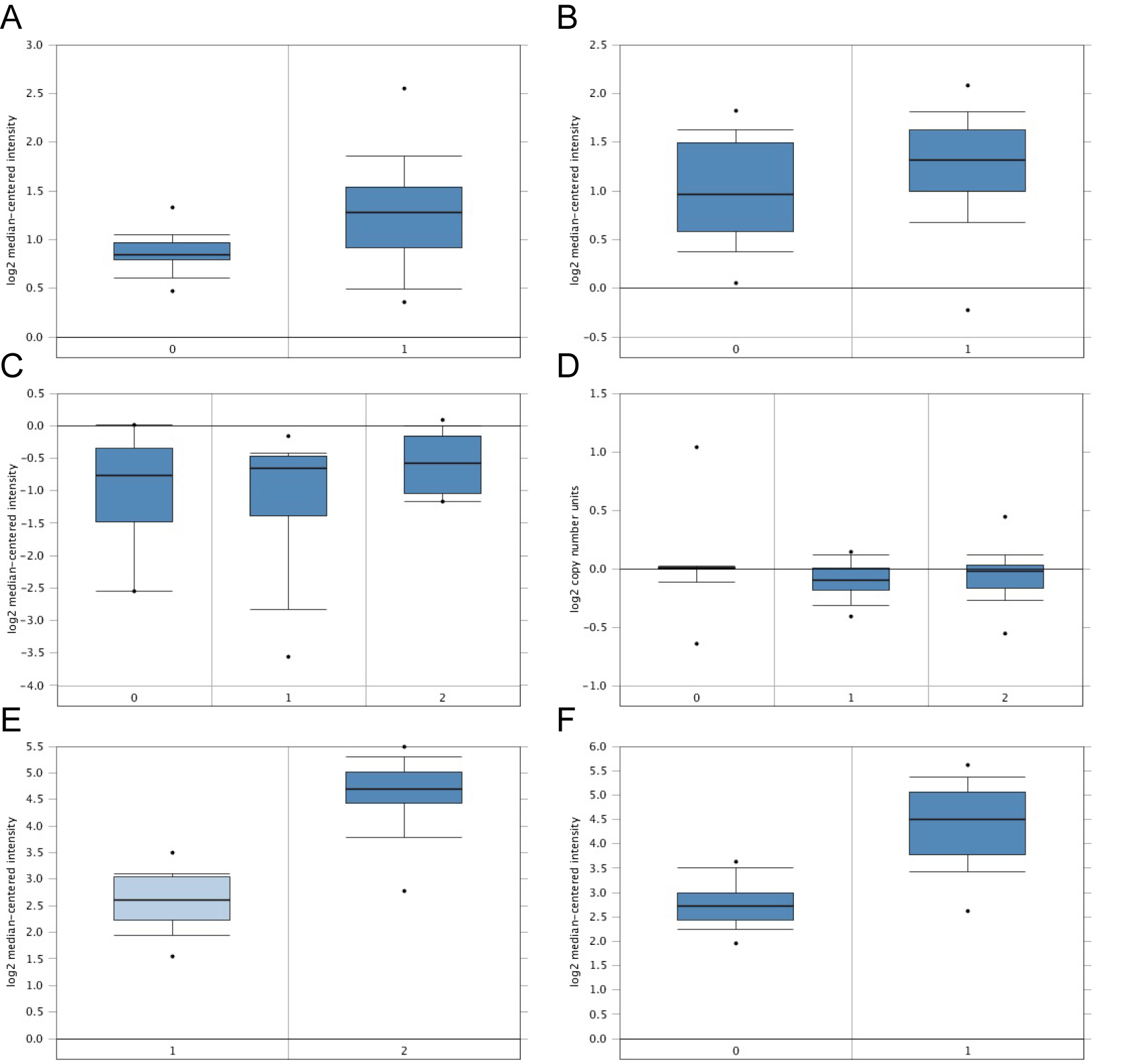


Supplementary Figure S5


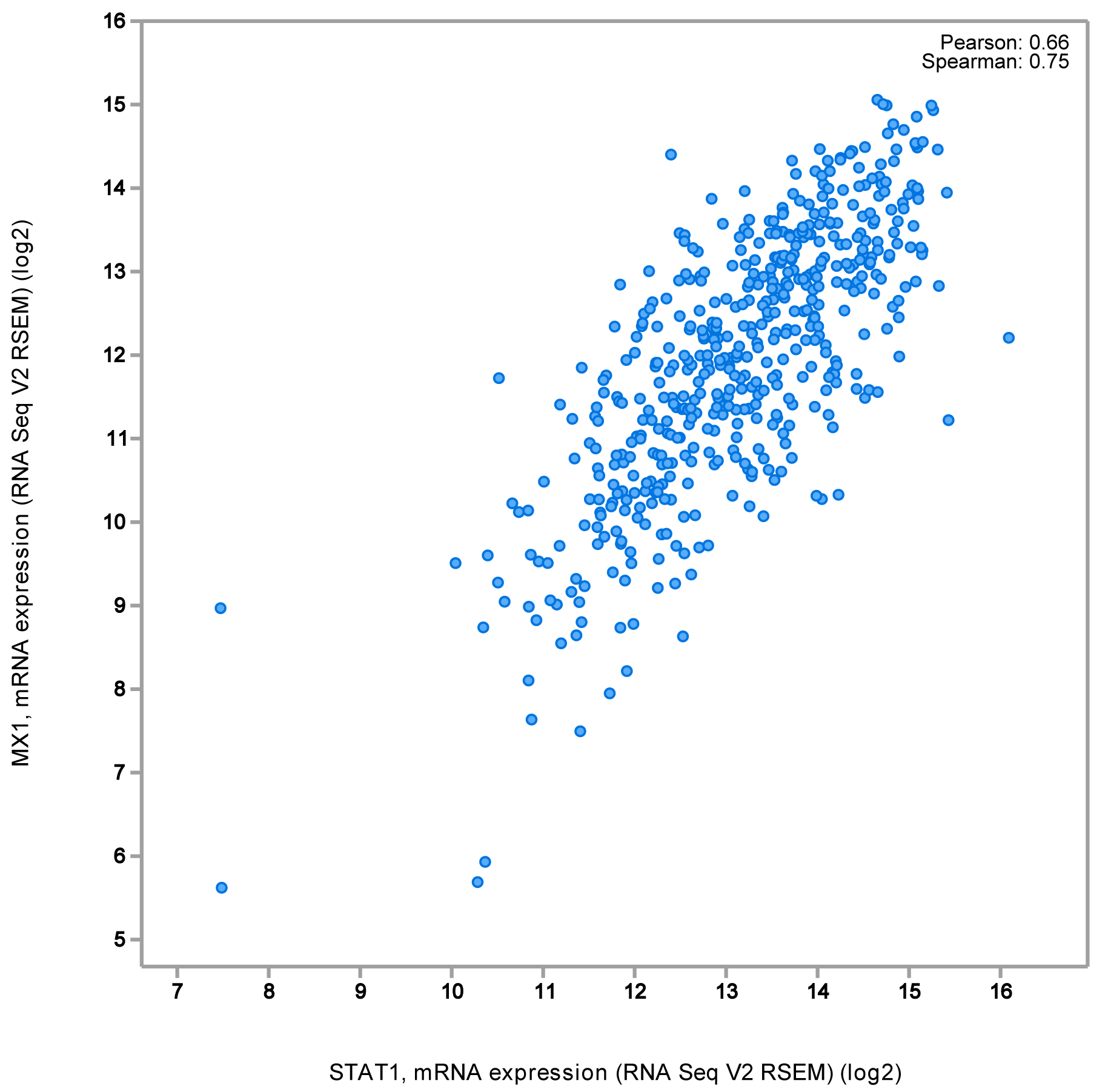


Supplementary Figure S6


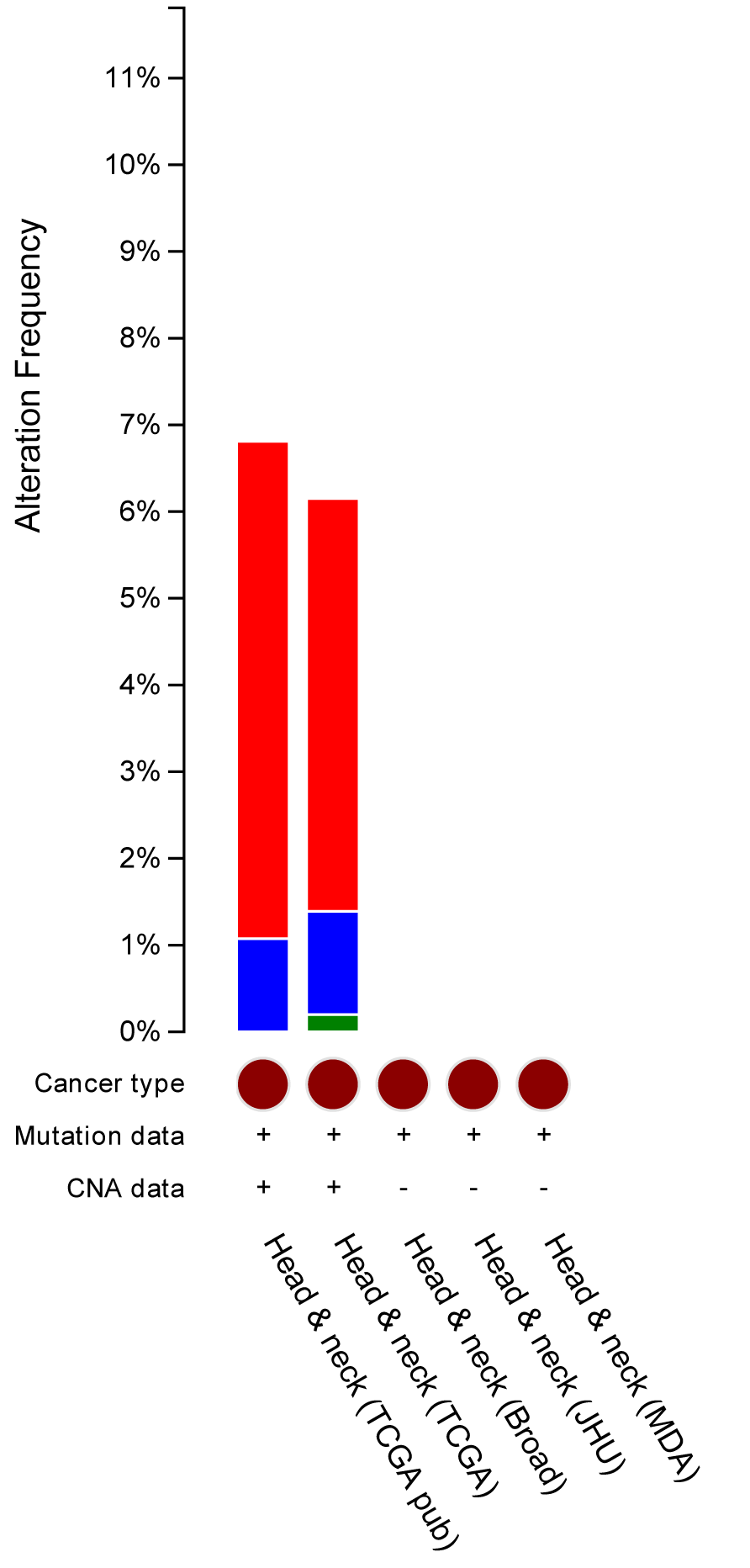


Supplementary Figure S7


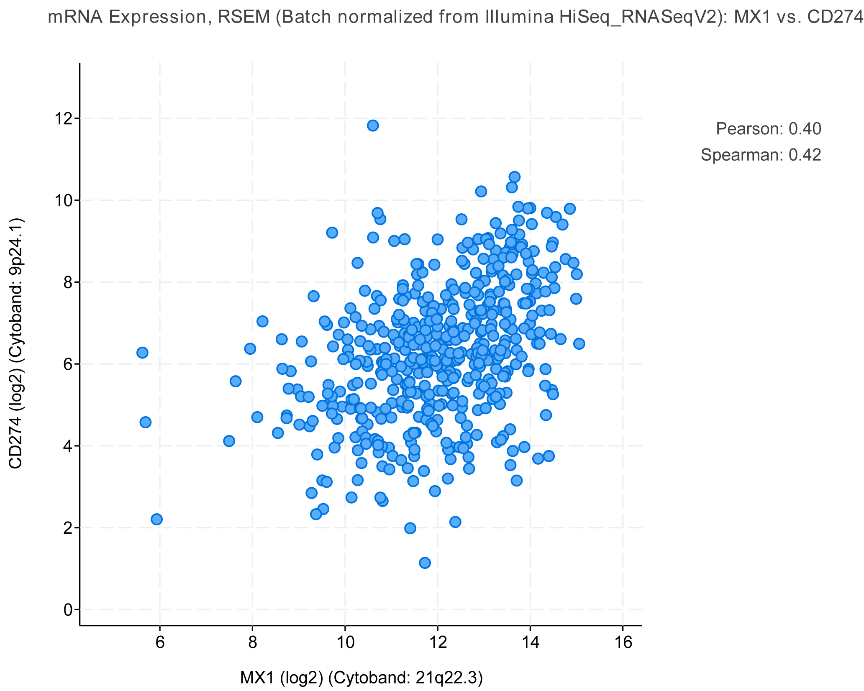


Supplementary Figure S8


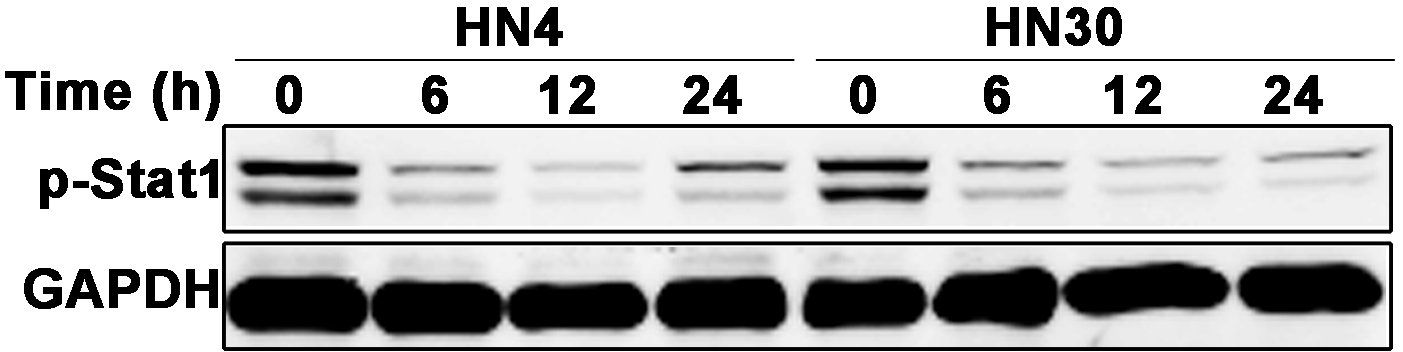


Supplementary Figure S9


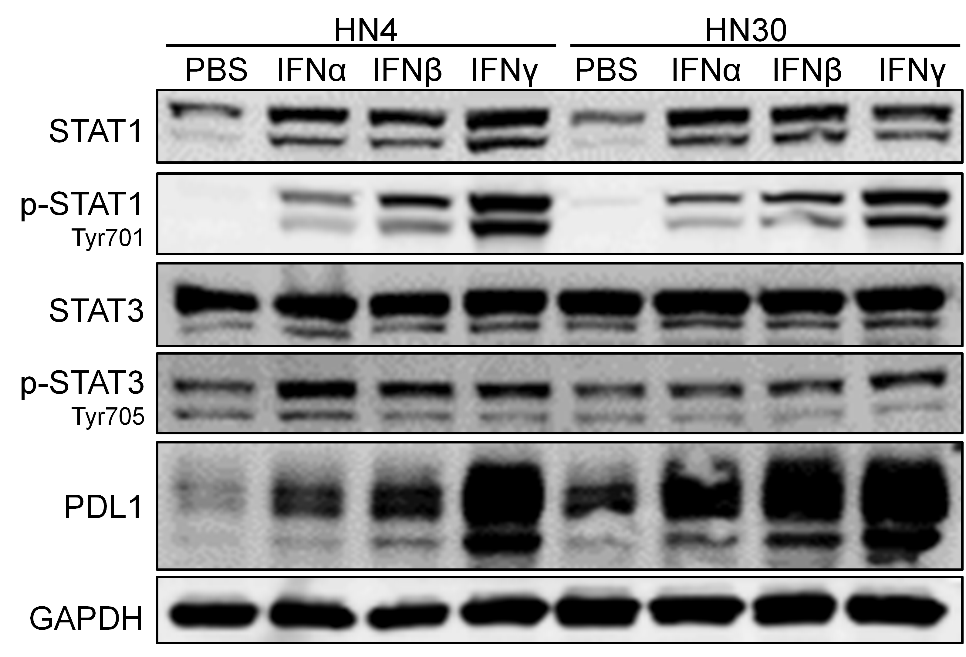


Supplementary Figure S10


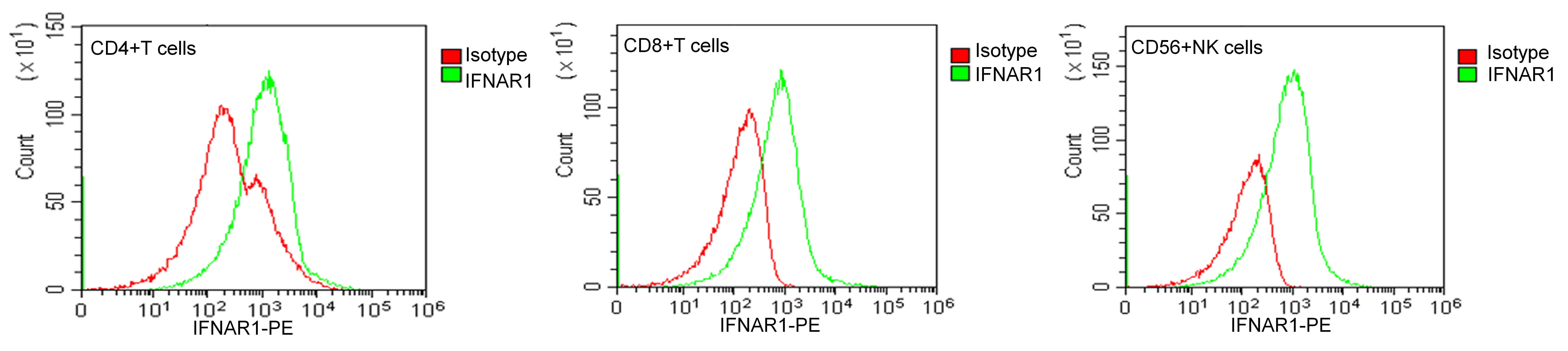


Supplementary Figure S11


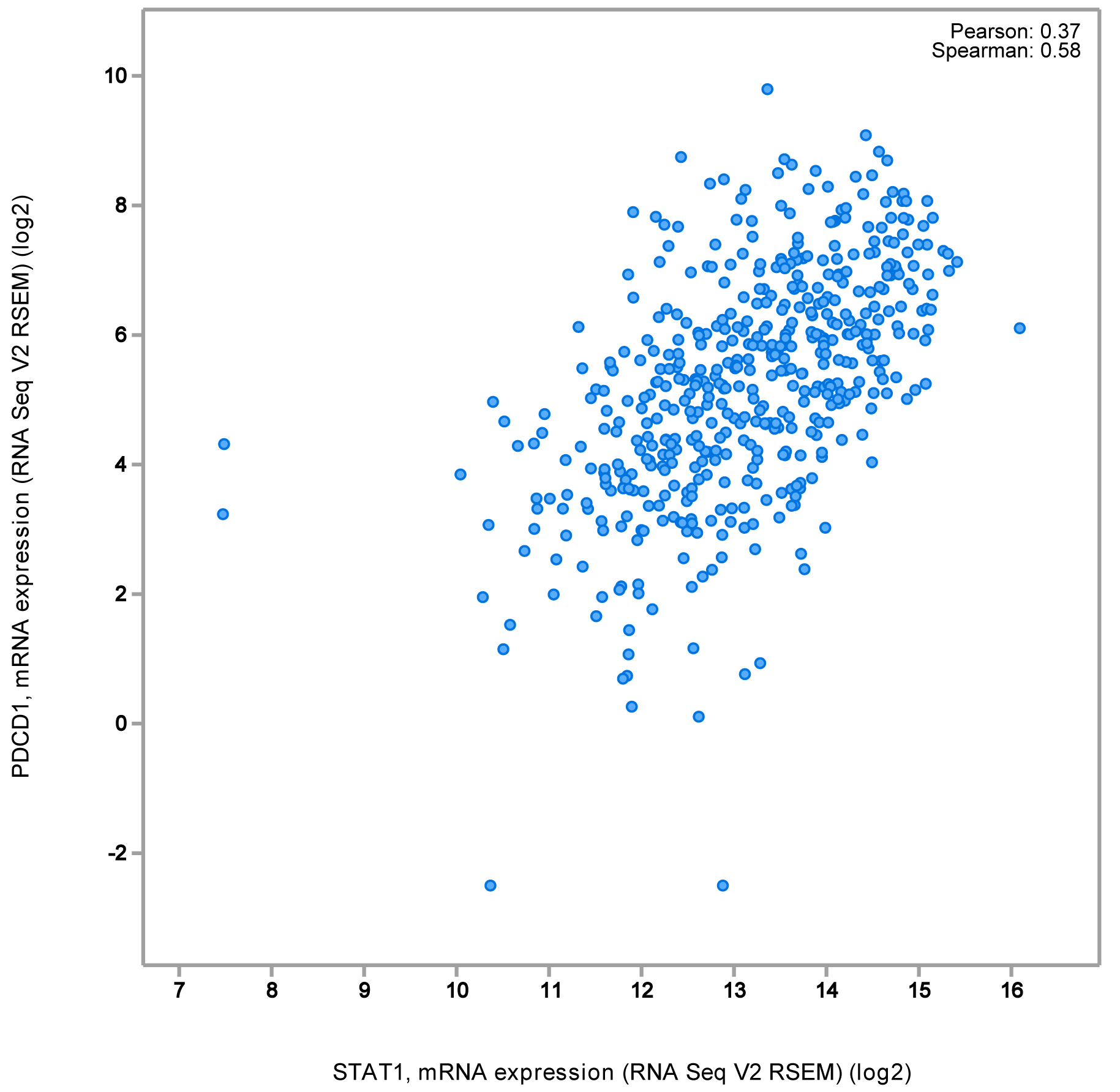


Supplementary Figure S12


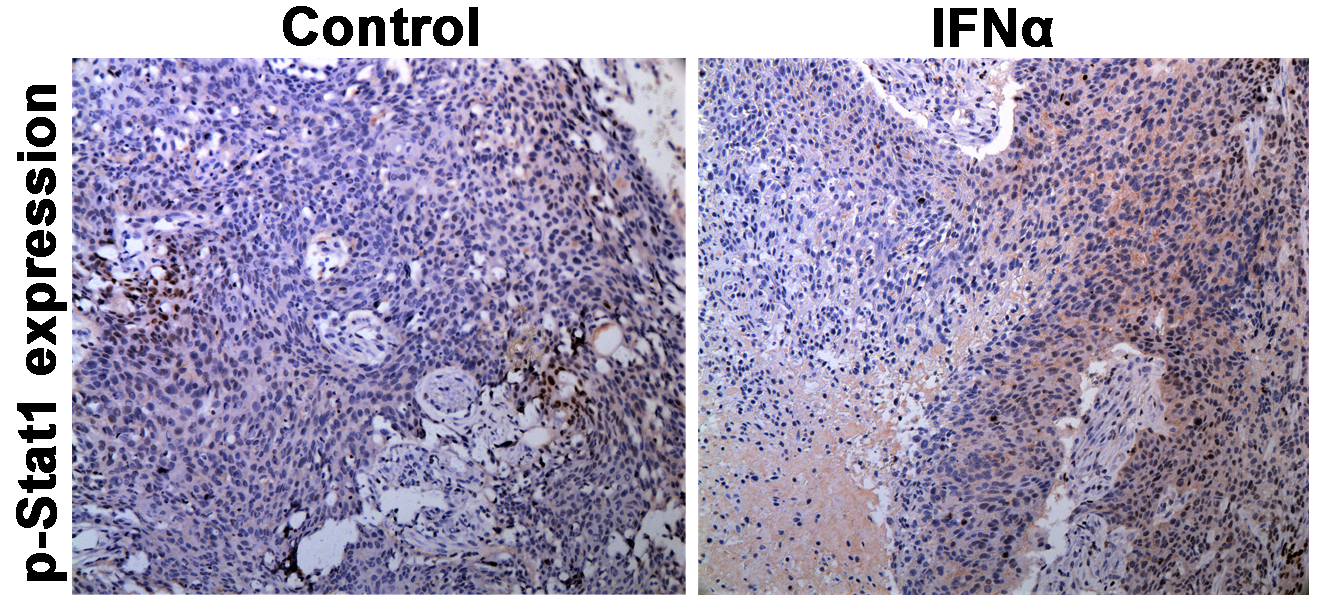

Supplement: Supplementary file 1 — Supplementary Tables and Figures [file 41416_2018_352_MOESM1_ESM.docx]
